# Prevalence of anti-HBc and anti-HBs among blood donors in Guangzhou: implications for HBV screening strategies in China

**DOI:** 10.1186/s12879-026-13487-0

**Published:** 2026-05-06

**Authors:** Zhengang Shan, Min Wang, Ru Xu, Fenfang Liao, Qiao Liao, Jieting Huang, Bochao Liu, Huishan Zhong, Yongshui Fu, Huaqin Liang, Xia Rong

**Affiliations:** 1 Institute of Blood Transfusion and Hematology, Guangzhou Blood Center, Guangzhou Medical University, Guangzhou, Guangdong 510095 China; 2 The Key Medical Laboratory of Guangzhou, Guangzhou, Guangdong 510095 China

**Keywords:** Anti-HBc screening, Guangzhou blood donors, Anti-HBs testing

## Abstract

**Background & aims:**

In China, blood donations are routinely screened for both HBsAg and HBV DNA to prevent transfusion-transmitted hepatitis B virus (HBV) infection. However, the benefit of implementing universal anti-HBc screening to intercept occult HBV infection remains debated, primarily due to concerns over donor loss and cost-effectiveness. This study aimed to assess the prevalence of anti-HBc among blood donors in Guangzhou and to evaluate whether the current HBV screening strategy in China requires further optimization.

**Methods:**

In this study, we enrolled 25,056 voluntary blood donors who tested negative for both HBsAg and HBV DNA. All samples were first screened for anti-HBc using ELISA. A subset of positive samples was then retested with an electrochemiluminescence (ECL) assay. Subsequently, quantitative anti-HBs testing was performed on samples that were positive by both assays, and those that were anti-HBs-negative underwent HBV RNA testing.

**Results:**

The initial anti-HBc-positive rate was 27.1% (6795/25,056), which exhibited a strong age-dependent increase but showed no association with gender. Of the 1343 samples that underwent retesting, 82.1% (1102/1343) were confirmed positive. Among the double-positive samples, 91.2% (990/1085) were also anti-HBs positive, and 53.5% (580/1085) presented with anti-HBs levels ≥ 200 IU/L. No statistically significant difference in anti-HBs levels was observed between genders or across age groups. Furthermore, HBV RNA was not detected in any of the 95 anti-HBs-negative samples tested.

**Conclusion:**

The high prevalence of anti-HBc, coupled with a substantial proportion of donors having protective levels of anti-HBs, suggests that the incremental benefit of universal anti-HBc screening in Guangzhou’s current donor population may be limited. While implementing such assays is not considered feasible at present, future declines in prevalence of HBV infection may warrant a re-evaluation and optimization of the screening strategy.

## Introduction

Hepatitis B virus (HBV) infection continues to pose a significant global public health challenge, particularly in regions with high endemicity [1]. According to the World Health Organization (WHO), an estimated 257 million individuals are chronic carriers of HBV, representing approximately 3.5% of the world’s population [2]. Consequently, HBV has historically been one of the most frequently detected blood-borne pathogens in transfusion medicine [3]. To prevent transfusion-transmitted HBV, blood donations in China are routinely screened for both HBV surface antigen (HBsAg) and HBV DNA. The detection of viral DNA is performed using automated platforms, either through minipool nucleic acid testing (MP-NAT) of six samples or individual donor nucleic acid testing (ID-NAT). Currently, blood establishments across China broadly adhere to unified operational standards, with the main differences primarily in the reagents employed [4].

Occult HBV infection (OBI) is defined as chronic HBV infection in the absence of detectable HBsAg [5]. It typically involves very low and often intermittent viremia. This characteristic makes OBI difficult to detect through routine blood screening. Even individual donation nucleic acid amplification testing cannot fully eliminate the risk of OBI transmission [6, 7]. Previous studies indicated that transfusion from donors with OBI were estimated to cause HBV infection in 8% to 29% of recipients, thereby representing a silent yet substantial threat to blood safety [8]. Among the various serological markers associated with HBV infection, antibodies to the hepatitis B core antigen (anti-HBc or HBcAb) are regarded as a reliable diagnostic indicator of prior exposure to the virus [1]. Anti-HBc screening was implemented in some developed countries, which was prompted by several cases of transfusion-transmitted HBV infection by donors with undetectable HBsAg and undetected HBV viremia [9]. However, the differentiation between truly resolved HBV infection, OBI with low intermittent viremia, and false-positive anti-HBc reactivity is complex due to inherent limitations in the assay [10–13]. To address this, a refined screening algorithm incorporating levels of antibodies to the hepatitis B surface antigen (anti-HBs or HBsAb) has been adopted in these settings: the eligibility of anti-HBc-positive donors with anti-HBs levels above 100 or 200 IU/L was maintained [9, 14].

At present, implementing universal anti-HBc screening in countries with potentially high burden of OBI is deemed impractical, as it could lead to a disproportionate loss of donors and subsequently jeopardize the national blood supply. Even in low-endemicity countries, concerns have been raised that anti-HBc screening might result in a substantial reduction in otherwise eligible donors [14]. Whether the number of intercepted infectious, intermittently viremic donations outweighs the loss of anti-HBc-positive, noninfectious donors remains controversial [14]. China has not yet implemented anti-HBc screening, largely due to its historically high HBV prevalence, where the sero-prevalence of HBsAg once approached 10% [15]. However, over the past few decades, China has witnessed a remarkable decline in HBV infection rates and is now considered a medium-endemic country for HBV, with an estimated HBsAg prevalence of 3% in the general population as of 2021 [15]. In Guangzhou — a major city in southern China — the sero-prevalence of HBsAg among blood donors was reported as 38 per 10,000 in 2023 [16]. In light of these epidemiological shifts, evaluating the cost-effectiveness of introducing anti-HBc screening has become increasingly relevant.

Although anti-HBc screening has been adopted in numerous countries, considerable heterogeneity persists globally in screening and confirmatory algorithms, as well as in follow-up strategies for anti-HBc-positive and OBI donors. This study aims to investigate the prevalence of anti-HBc in the current blood donor population in Guangzhou and preliminarily evaluate the added benefit of anti-HBc screening in intercepting potentially infectious blood from donors with OBI. The ultimate goal is to explore whether the existing HBV screening algorithm can be optimized without compromising the safety and sustainability of the local blood supply.

## Methods

### Study participants

**Fig. 1:**
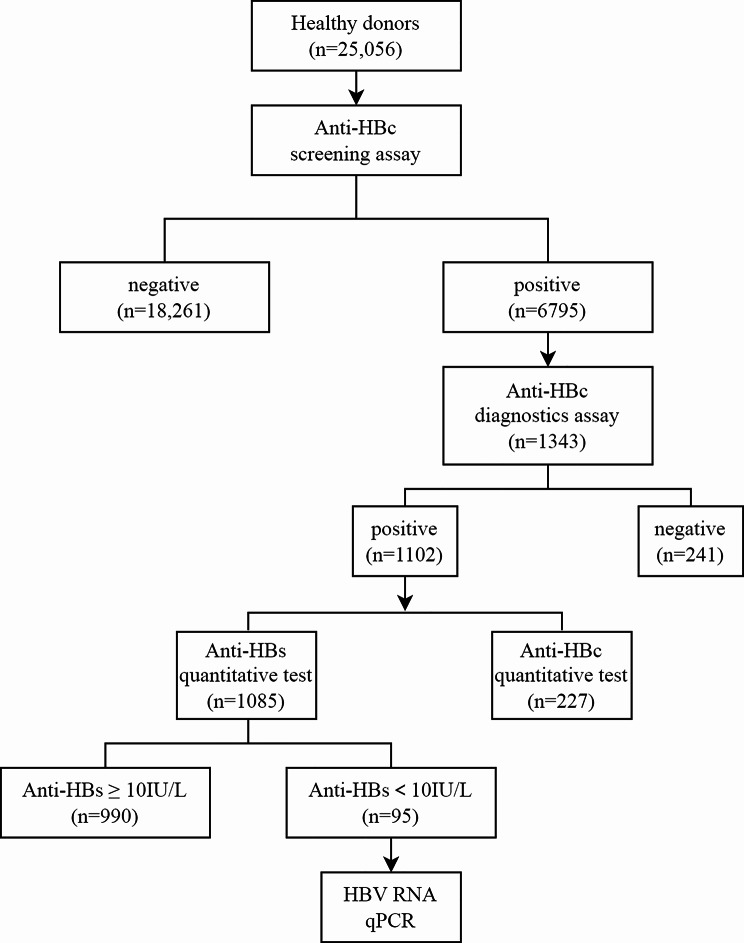
Study testing flowchart

This study was conducted at Guangzhou Blood Center in Guangdong Province, China. A total of 25,056 healthy volunteer blood donors were recruited, who donated blood between April and June 2025. In China, routine blood screening covers hepatitis B virus, hepatitis C virus, human immunodeficiency virus, and *Treponema pallidum*. Each pathogen was tested in parallel using two serological screening kits from different manufacturers, with nucleic acid testing (NAT) additionally performed for the three viruses. Healthy blood donors are defined as individuals with negative results for both serological screening and nucleic acid testing. Donors with two or more positive results are subject to permanent deferral, whereas those with a single positive result are temporarily deferred and eligible for retesting after a specified interval. With specific regard to HBV routine screening, serological assays are performed using HBsAg ELISA kits from Wantai (Beijing, China) and InTec (Xiamen, China), while HBV DNA testing is conducted on either the Roche cobas 5800 system or the Grifols Procleix Panther system. To ensure confidentiality, all collected samples and data were anonymized. The flow chart in Fig. 1 maps out the testing performed in this study.

### Sample collection

Venous blood (4–5 mL) was collected from each donor into sterile EDTA tubes labeled with a unique donor identification number and temporarily stored at 4 °C. After routine screening, plasma was separated by centrifugation and transferred into microcentrifuge tubes for subsequent analysis. All plasma samples were stored at -20 °C for no longer than 72 h.

### Anti-HBc and anti-HBs testing

All samples were screened for anti-HBc using qualitative HBcAb ELISA Kit (Wantai, China). Subsequently, the last subset of samples yielding positive results was retested with qualitative Elecsys Anti-HBc II (Roche, Germany). Samples that tested positive in this diagnostic assay underwent further testing for anti-HBs using the quantitative Elecsys Anti-HBs II (Roche, Germany; LoD: 2 IU/L). In addition, a small number of the confirmed anti-HBc-positive samples were also subjected to quantitative anti-HBc testing (Wantai, China; LoD: 0.1 IU/mL).

### RNA extraction and real-time PCR quantification

Viral RNA was isolated from 300 µL of plasma (anti-HBc-positive/anti-HBs-negative) using magnetic beads (Hotgen, China) and subsequently quantified using fluorescence probes (Hotgen, China; LoD: 50 copies/mL). Extracted RNA was frozen at -80 °C.

### Data analysis

The collected data, including demographic details and donation records, were organized and tabulated using Microsoft Excel (Office 2016). Random sampling was conducted using Excel’s RANDBETWEEN function. Statistical analyses were conducted with IBM SPSS (version 22), employing the chi-square test, M-W test and K-W test to evaluate differences, where a P-value below 0.05 indicated statistical significance.

## Results

### Demographic characteristics of blood donors

The demographic characteristics of the 25,056 participants are summarized in Table 1. The cohort was relatively young, with the largest group being donors aged 18–25 years, accounting for 45.5% (*n* = 11,411). The 26–35 age group constituted another 24.2% (*n* = 6070), meaning that donors under 36 years old collectively represented over two-thirds of the cohort. Only 1.0% (*n* = 263) fell within the 56–60 age range — a group consisting exclusively of repeat donors, as initial blood donation in China is permitted only for individuals under 56 years of age. Regarding gender distribution, males accounted for 62.4% of participants.

**Table 1:** Participants’ demographic characteristics

		Number of blood donors (%)
Gender	Men	15,626 (62.4)
	Women	9430 (37.6)
Age	18-25	11,411 (45.5)
	26-35	6070 (24.2)
	36-45	4786 (19.1)
	46-55	2526 (10.1)
	56-60	263 (1.0)
Total		25,056

### Anti-HBc screening

**Fig. 2:**
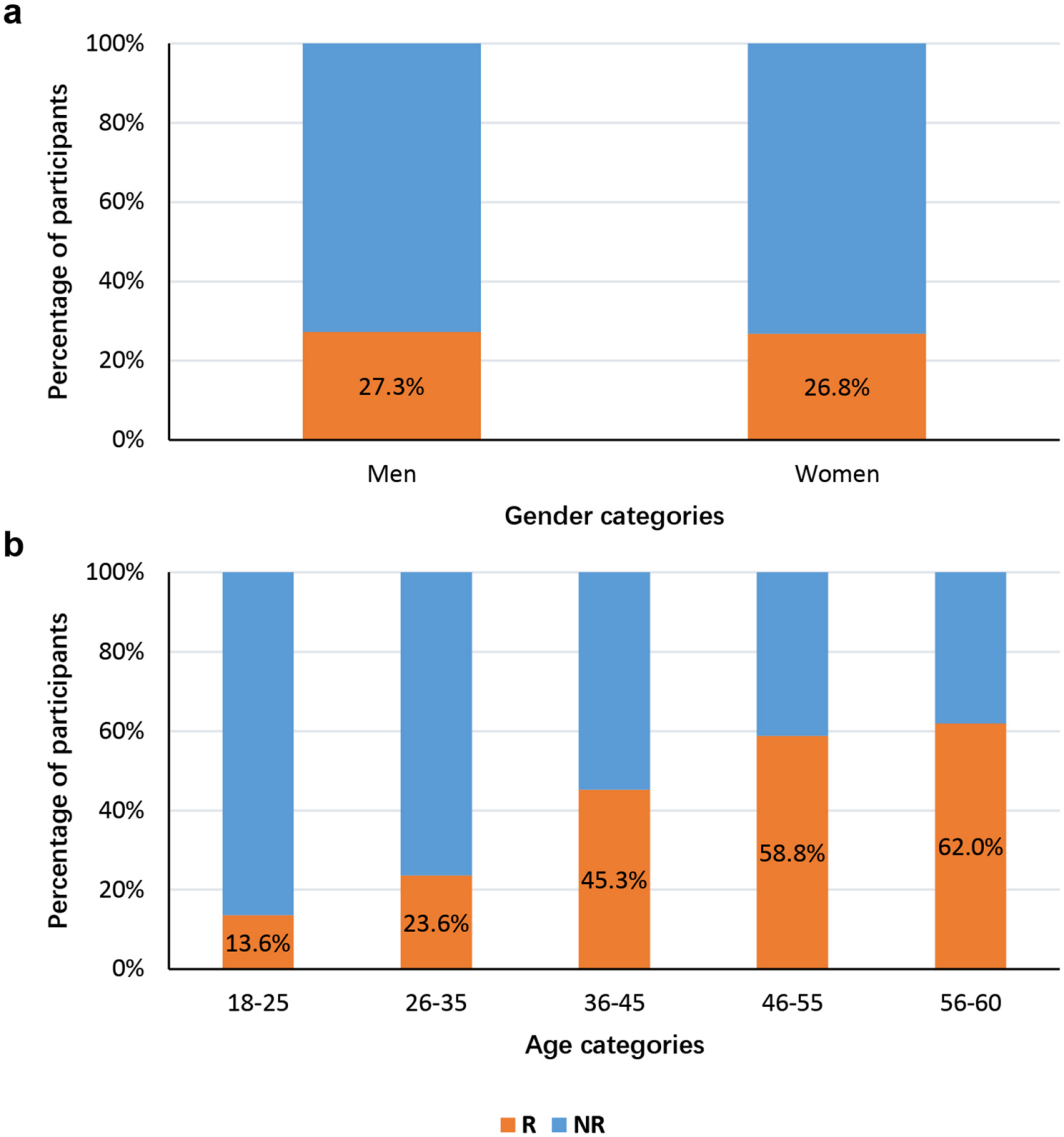
Anti-HBc sero-positivity by donor gender and age. **a**) Comparison between men and women; no statistically significant difference was observed. **b**) Comparison across age groups; differences between consecutive groups were significant (*p* < 0.01) except between the two oldest groups. R: positive reaction; NR: negative reaction

After qualitative screening of 25,056 blood samples for anti-HBc, 27.1% (*n* = 6795) tested positive. The positive rate did not differ significantly between male and female donors (Fig. 2a). In contrast, a clear age-related trend was observed: the prevalence of anti-HBc increased markedly with age (*p* < 0.01), rising from 13.6% in the youngest group to more than 50% in donors aged over 45 years (Fig. 2b).

Of the initially positive samples, 1343 were retested using electrochemiluminescence (ECL), and 82.1% (*n* = 1102) remained positive. From these double-positive samples, 227 were randomly selected and further analyzed by quantitative anti-HBc assay. Results showed that 92.5% (*n* = 210) had anti-HBc levels below 100 IU/mL, with a median of 8.13 IU/mL.

### Anti-HBs testing

Of the 1102 confirmed anti-HBc-positive samples, 17 were excluded due to insufficient plasma volume for further testing. The remaining 1085 samples all underwent quantitative anti-HBs detection. As shown in Table 2, 91.2% (*n* = 990) of the donors tested positive for anti-HBs, with over half exhibiting levels greater than 200 IU/L. No statistically significant difference in anti-HBs levels was observed between different genders or across age groups.

**Table 2:** Anti-HBs positive rates by donor gender and age groups

		Anti-HBc	Anti-HBs	Anti-HBs	Median
		double-positive	≥10 IU/L^a^	≥200 IU/L^b^	(IU/L)
Gender	Men	704	646 (91.8%)	373 (53.0%)	242.8
	Women	381	344 (90.3%)	207 (54.3%)	254.6
Age	18-25	26	22 (84.6%)	10 (38.5%)	144.4
	26-35	209	197 (94.3%)	115 (55.0%)	288.5
	36-45	491	450 (91.6%)	280 (57.0%)	288
	46-55	335	299 (89.3%)	162 (48.4%)	184.9
	56-60	24	22 (91.7%)	13 (54.2%)	453
Total		1085	990 (91.2%)	580 (53.5%)	245.6

^a^ The assay cut-off for anti-HBs is 10 IU/L

^b^ Anti-HBs ≥ 200 IU/L is generally accepted as the threshold for retaining anti-HBc-positive blood donors

Only 210 samples had available quantitative data for both anti-HBs and anti-HBc. Among these samples, we compared anti-HBc levels across different anti-HBs statuses. The results showed that anti-HBc levels appeared higher in anti-HBs-positive donors, but no statistically significant difference was observed (Table 3).

**Table 3:** Quantitative detection of anti-HBc grouped by anti-HBs levels

	Number of samples^b^	Median	Maximum	IQR
		(IU/mL)	(IU/mL)	(IU/mL)
Anti-HBs negative	18	5.4	239.32	51.93
Anti-HBs positive (<200 IU/L)	80	7.74	812.95	33.47
Anti-HBs positive (≥200 IU/L)^a^	112	9.08	817.16	49.16

Abbreviations: IQR, Interquartile Range

^a^Anti-HBs ≥ 200 IU/L is generally accepted as the threshold for retaining anti-HBc-positive blood donors

^b^Among the 1,085 anti-HBc double-positive samples, only 210 had available quantitative results for both anti-HBc and anti-HBs

### Detection of HBV RNA

All 95 samples that were anti-HBc positive but anti-HBs negative were tested for HBV RNA by real-time quantitative PCR (qPCR) and none yielded a positive result.

### Records of donor deferral

A retrospective analysis of donation records was conducted, comparing 1102 donors who were double-positive for anti-HBc with 2848 randomly selected anti-HBc-negative donors. In the double-positive group, 18 donors (1.6%) had a prior deferral due to NAT positivity. All had their eligibility reinstated after testing negative on ID-NAT six months later. A similar history was found in 11 donors (0.4%) of the negative group. Given that early blood screening protocols did not include nucleic acid testing, these data were considered insufficiently rigorous for formal statistical analysis.

## Discussion

According to the International Society of Blood Transfusion (ISBT), more than 13 countries have implemented universal anti-HBc screening. Among surveyed blood establishments, the overall anti-HBc prevalence was 0.42%, with individual rates ranging from 0.01% to 5.18% [9]. In contrast, our study found that more than a quarter of blood donors tested positive for anti-HBc. If all anti-HBc positive donors were to be permanently deferred without confirmatory or supplementary testing, Guangzhou could lose over 25% of its blood supply — a scenario that would severely compromise the sustainability of the local blood services.

Regarding the effectiveness of anti-HBc screening, most assays exhibit a relatively high rate of false positivity [17, 18]. Blood establishments have adopted varied strategies to confirm initially positive anti-HBc results: some perform no confirmation, some retest using the same assay, and others employ a secondary anti-HBc test from a different manufacturer [9]. It has been estimated that approximately 20% of donors with initially positive but unconfirmed anti-HBc results may not require permanent deferral [18]. In the present study, 17.9% of donors who were initially positive by ELISA tested negative by ECL, a proportion very close to the aforementioned estimate.

Anti-HBc positive donations with high anti-HBs levels (e.g., > 100 or 200 IU/L) are generally considered acceptable under the assumption that circulating anti-HBs can neutralize potential HBV infectivity. This viewpoint is supported by the absence of documented transfusion-transmitted HBV infections from DNA-positive donations with anti-HBs levels exceeding 100 IU/L [9]. In our study, 53.5% of anti-HBc double-positive donors presented with anti-HBs levels above 200 IU/L. If such a threshold were adopted as the deferral criterion, the proportion of donors lost would decrease from 27.1% to 10.3%. However, even this reduced loss remains operationally unsustainable, especially in areas like Guangzhou where blood supply is already tight. Moreover, the overall cost of screening would rise substantially. For instance, preliminary cost projections at Guangzhou Blood Center suggest that the introduction of both anti-HBc and anti-HBs screening would require more than 5 million RMB in additional annual expenditure.

The extent to which the risk of transfusion-transmitted HBV would increase in the absence of anti-HBc screening remains controversial, as relevant research data are currently inadequate. According to the annual Serious Hazards of Transfusion (SHOT) report from England, three cases of transmission were documented prior to the implementation of universal anti-HBc screening [19]. These cases involved two anti-HBc-positive donors with OBI whose viral loads were below the limit of detection of MP-NAT, resulting in HBV transmission to three recipients [19]. No similar transmissions have been reported since the introduction of anti-HBc screening. In Guangzhou clinical records, we have not identified any confirmed cases of HBV transmission from OBI donors. This local finding is consistent with international observations, where such cases are also unreported in countries that do not perform universal anti-HBc screening. Based on our data, 91.2% of anti-HBc-positive blood donors are also positive for anti-HBs. This high coexistence rate may be a key factor underlying the absence of transfusion-transmitted hepatitis B cases in Guangzhou despite not performing anti-HBc screening. However, it should be noted that HBV transmissions from OBI donors are likely underreported worldwide due to several factors, including the frequent asymptomatic nature of HBV infection, the absence of systematic donor lookback programs, and the possibility of viral clearance by the time such investigations are conducted [20].

It has been established that both HBV DNA and RNA can be detected in the peripheral blood of infected individuals, with RNA sometimes persisting even when DNA is undetectable [21]. Accordingly, we performed HBV RNA testing on samples that were anti-HBc positive but anti-HBs negative, yet no positive results were observed. This could be attributable to the limited sample size. As demonstrated in a previous study, the detection rate of HBV RNA is generally lower than that of HBV DNA [21]. Consequently, the RNA positivity rate in our NAT-negative cohort was expectedly minimal, making the absence of any positive findings entirely plausible given the limited sample size. Another contributing factor may be the relatively low sensitivity of the assay used in our study, which had a detection limit of 50 copies/mL for HBV pre-genome RNA (pgRNA). In comparison, the ID-NAT implemented on the cobas 5800 system at Guangzhou Blood Center achieves a significantly lower HBV DNA detection limit of 1.4 IU/mL (≈ 7.84 copies/mL). A previous study reported that the average anti-HBc level in OBI individuals was significantly higher than in those with past HBV infection, with an optimal cutoff of 6.6 IU/mL (AUROC = 0.723, sensitivity: 60.7%, specificity: 75.3%) [22]. In our study, blood donors who were anti-HBs negative but anti-HBc positive exhibited a median anti-HBc level of 5.4 IU/mL, indicating that most of them had likely resolved HBV infection. However, due to the invasive nature of liver biopsy, hepatic tissue DNA testing was not performed. Consequently, anti-HBc quantification alone was insufficient for definitive diagnosis.

In the present study, anti-HBc positivity showed no gender association but exhibited a significant correlation with age, with markedly reduced rates in younger donors. This trend primarily stems from the widespread implementation of hepatitis B vaccination in China since 1992. Enhanced vaccination coverage and improved healthcare infrastructure have driven the rapid decline in HBV prevalence. Nevertheless, vaccination history was not incorporated into our analysis. Several other limitations should also be noted. Firstly, the relatively small number of samples with anti-HBc quantification restricted our ability to conduct further subgroup or trend analyses. Secondly, as a cross-sectional study, it lacked follow-up data for key subgroups, such as donors with isolated anti-HBc positivity, which may lead to inaccuracies in the grouping of blood donors. Finally, our findings are limited in generalizability by the absence of collaborative studies with blood establishments domestically and abroad.

## Conclusion

Currently, the potential benefit of implementing universal anti-HBc screening in Guangzhou appears to be limited, given the high baseline prevalence of anti-HBc and the relatively frequent co-presence of anti-HBs among blood donors. Nevertheless, with the projected decline in HBV prevalence rates, the optimization of blood screening algorithms will remain a crucial issue in transfusion medicine. Moreover, in alignment with the WHO’s viral hepatitis elimination goals, it is recommended that Chinese blood establishments participate in global research cooperation on anti-HBc screening and investigate cost-effective approaches in resource-constrained settings.

## Data Availability

The raw data supporting the conclusions of this article will be made available by the authors on request.

## Abbreviations

HBV: Hepatitis B virus
HBsAg: Hepatitis B surface antigen
Anti-HBc/HBcAb: Antibodies to hepatitis B core antigen
Anti-HBs/HBsAb: Antibodies to hepatitis B surface antigen
ELISA: Enzyme-linked immunosorbent assay
ECL: Electrochemiluminescence
OBI: Occult hepatitis B infection
DNA: Deoxyribonucleic acid
RNA: Ribonucleic acid
NAT: Nucleic acid testing
EDTA: Ethylenediamine tetraacetic acid
PCR: Polymerase chain reaction
AUROC: Area under receiver operating characteristic curve
